# Progress in research on the role of circular RNAs in lung cancer

**DOI:** 10.1186/s12957-018-1515-2

**Published:** 2018-11-06

**Authors:** Yang Chen, Shuzhen Wei, Xiyong Wang, Xiaoli Zhu, Shuhua Han

**Affiliations:** 1 Department of Respiratory, Zhongda Hospital, Southeast University, Nanjing, China; 2 Medical School of Southeast University, Nanjing, China

**Keywords:** Lung cancer, Circular RNA, Endogenous regulation, Biological diagnosis, Biomarker

## Abstract

**Background:**

Circular RNA (circRNA), as a covalently closed circular RNA molecule, is widely present, which is recognized as a competing endogenous RNA. A large number of differentially expressed circRNAs have been identified and are recognized as potential biomarkers for the diagnosis of tumors.

**Main body:**

CircRNAs play an important role in the regulation of cell signaling pathways. The main biological functions of circRNAs include acting as miRNA sponges, regulating the transcription of the parental genes, and acting as adapters to regulate the interactions between proteins and encoding proteins. Compared with normal tissues, there are differentially expressed circRNAs in lung cancer tissue, and the expression levels of circRNAs are correlated with clinicopathological features of lung cancer. Their roles in pathway regulation are described, and the diagnostic and prognostic values are further evaluated.

**Conclusion:**

In lung cancer, circRNAs participate in the proliferation, migration, and invasion, acting as a competitive endogenous RNA. Differentially expressed circRNAs may serve as non-invasive diagnostic markers for lung cancers. Further investigation of the roles of circRNAs in the pathogenesis and regulatory pathways is conducive to the development of novel approaches for the diagnosis and accurate treatment of lung cancers.

## Background

In the 1970s, Sanger et al. examined viroids by electron microscopy and discovered that the viroids were single-stranded RNA molecules with a covalently closed circular structure and high thermal stability [1]. In the early days of the discovery of circRNAs, due to the limitation of the detection techniques, most circRNAs were expressed in only a few cell types and at low abundance. With the development of RNA sequencing and bioinformatics technologies in recent years, circular RNAs were found to be stable and prevalent in a variety of species and tissues, with cell phenotype specificity and tissue developmental stage specificity. Xu et al. analyzed six types of normal human tissues (colon, heart, kidney, liver, lung, and stomach tissues) based on RNA-seq data and detected at least 1000 circRNAs in each tissue [2]. Approximately 36.97–50.04% of the circRNAs exhibited tissue-specific expression. For example, 1224 circRNAs were identified in adult normal lung tissues, among which 452 were specifically expressed.

The regulatory mechanism of circular RNAs has been further explored. Some of the circRNAs play an endogenous regulatory role by acting as a sponge to adsorb microRNAs (miRNAs). These circRNAs affect the functions of target genes downstream of the miRNAs, thereby participating in tumor development and progression. To date, a large number of differentially expressed circRNAs have been identified in esophageal cancer, gastric cancer, and colon cancer and are recognized as potential biomarkers for diagnosis. Lung cancer is a malignancy with the highest mortality rate worldwide [3]. The diagnosis and treatment of lung cancer significantly influence patient prognosis. At present, the 5-year survival rate of lung cancer patients is merely 17.7% [4]. The survival rate is significantly increased in patients with early-stage lung cancer compared with patients with advanced lung cancer (the 5-year survival rate of patients with early-stage lung cancer was 55.6%, whereas the 5-year survival rate of patients with advanced lung cancer was 4.5%) [5]. Therefore, early detection of lung cancer is crucial. The biological methods for efficient diagnosis of lung cancer is worthy of further exploration. Zhao et al. carried out a high-throughput circRNA microarray to investigate the expression profile of circRNAs in tumor tissues and adjacent normal tissues from four patients with early lung adenocarcinoma [6]. It was found that 356 circRNAs were differentially expressed. Two hundred four circRNAs were upregulated, and 152 circRNAs were downregulated in tumor samples. The discovery of lung cancer-related circRNAs has provided novel ideas for the diagnosis and treatment of lung cancer. By reviewing the biological functions and regulation mechanisms of circRNAs as well as the lung cancer-related pathways regulated by circRNAs, this paper further expounds the potential value of circRNAs as diagnostic and prognostic markers or therapeutic targets for lung cancer.

## Main text

### The functions of circRNAs

To date, numerous studies have assessed circRNAs. The biological functions of circRNAs have gradually been recognized by scholars. Currently, the known functions of circRNAs include acting as miRNA sponges, regulating the transcription of the parental genes, and acting as adapters to regulate the interactions between proteins and encoding proteins.

#### CircRNAs act as a miRNA sponge

CircRNAs could function as a miRNA sponge to regulate the gene expression. CDR1as is an antisense transcript of cerebellar degeneration-related protein 1 (CDR1) [7] that contains 63 conserved miR-7 binding sites. After binding to miR-7, CDR1as inhibits the function of miR-7 and exerts a negative regulatory effect. As a competitive endogenous RNA (ceRNA), circRNA can compete with miRNA. miRNA is usually combined with argonaute 2 (AGO2) protein to form RNA-induced silencing complex (RISC), thus regulating the expression of target genes. Because AGO2 can combine with circRNAs and miRNAs, the RNA-protein complex can be precipitated under the action of AGO2 protein antibody by RNA immunoprecipitation (RIP) experiment. Through sequencing RNAs combined with AGO2, the binding targets of miRNAs can be found. CircRNF13 [8] is a highly expressed circular RNA in lung cancer. The RIP experiment was used to explore the RNAs precipitated by AGO2 antibody and IgG antibody. It was found that circRNF13 and miR-93-5p were more obviously enriched in RNAs retrieved from the AGO2 antibody, which confirmed that circRNF13 and miR-93-5p could be directly combined, and circRNF13 could function as a sponge for miR-93-5p. However, Guo et al. found that circRNA-forming exons did not exhibit higher argonaute 2 (AGO2)-binding ability [9]. Therefore, they hypothesized that only certain circRNAs were able to act as sponges to adsorb miRNAs, whereas the majority of circRNAs did not have such function.

#### CircRNAs regulate the transcription of parental genes

CircRNAs are capable of regulating the transcription of parental genes. Intronic circRNAs (ciRNAs), which are formed from introns, contain a small number of miRNA binding sites and are virtually incapable of acting as miRNA sponges. However, ciRNAs could positively regulate RNA polymerase II (RNA Pol II) and promote the expression of maternal genes [10]. Exon-intron circRNAs (EIciRNAs), which are formed from exons and introns, interact with U1 small nuclear RNA (snRNA) through RNA-RNA binding, forming the EIciRNA-U1 snRNP complex. The complex promotes the expression of parental genes by affecting RNA Pol II [11].

#### CircRNA functions as an adapter between proteins

CircRNAs may exert their regulatory effects by binding to proteins. For example, circRNA derived from forkhead box O3 (circ-Foxo3) is related to cell senescence. circ-Foxo3 is highly expressed in senescent cardiomyocytes and is capable of inhibiting cell proliferation and cell cycle progression. circ-Foxo3 binds to cyclin-dependent kinases 2 (CDK2) and cyclin-dependent kinase inhibitor p21, forming the circ-Foxo3-p21-CDK2 ternary complex. The formation of the ternary complex reduces CDK2 activity, thereby further inhibiting cell cycle progression [12]. Similarly, circ-Foxo3 interacts with the anti-aging protein ID-1 (inhibitor of DNA binding 1), the transcription factor E2F1, and the anti-stress proteins FAK (focal adhesion kinase) and HIF1α (hypoxia-inducible factor 1-alpha) [13]. Such interactions block the entry of circ-Foxo3 into the nucleus and suppress the anti-aging and anti-stress effects of circ-Foxo3, resulting in cell senescence. The interactions between circRNAs and proteins expand the regulatory functions of circRNAs.

#### CircRNAs have protein translation functions

CircRNAs can be translated into proteins via a rolling circle mechanism, which not only provides repeated polypeptide sequences but also enhances polypeptide yields per unit of time as ribosomes do not need to bind repeatedly to the RNA template [14]. Rice yellow mottle virus consists of a covalently closed circular (CCC) RNA that can be directly translated into a 16-kD protein. CCC RNA contains one internal ribosome entry site (IRES) and two to three open reading frames (ORFs), which play important roles in the translation process [15]. Circular RNAs are rich in IRES and ORF elements. Of the 32,914 human exonic circRNAs included in the circRNADb database, 16,328 circRNAs contain ORFs that encode greater than 100 amino acids. Among the 16,328 circRNAs, 7170 contain IRES elements [16]. The presence of abundant IRES and ORF elements in circRNAs indicates that the translation of circRNAs may have a more general significance in human cells. CircRNAs participate in the regulation of tumors through translation into proteins. Yang et al. found that the circRNA derived from the F-box/WD repeat-containing protein 7 (FBXW7) gene (circ-FBXW7) encoded a 21-kDa protein FBXW7-185aa [17]. circ-FBXW7 and FBXW7-185aa were expressed at low levels in glioblastoma tissues, and a correlation between the expression level of circ-FBXW7 and the prognosis of patients with glioblastoma was noted. Functional studies revealed that upregulation of FBXW7-185aa in tumor cells inhibited cell proliferation and cell cycle progression, whereas knockdown of FBXW7-185aa promoted the malignant development of tumors. The translation of circRNAs and their regulatory role in tumor tissues may provide ideas for further research on circRNAs.

### Circular RNAs and lung cancer

Lung cancer-related studies reveal that circRNAs play an endogenous regulatory role in the development and progression of lung cancers and have potential diagnostic values (Table 1).

**Table 1:** Circular RNAs in lung cancer

CircRNA	Expression level	Function	Mechanism	Diagnostic and prognostic value	Reference
circFARSA	Upregulated in tissues and plasma	To promote cell migration and invasion	Sponge miR-330-5p and miR-326; upregulate fatty acid synthase	The area under the ROC curve was 0.71	[40]
CircRNA 100876	Upregulated in tissues	Unknown	Unknown	Higher expression level associated with lower overall survival	[26]
Hsa_circ_0013958	Upregulated in tissues, cells, and plasma	To promote cell proliferation and invasion and prevent apoptosis	Sponge miR-134; upregulate cyclin D1	The area under the ROC curve was 0.815	[27]
Circular RNA-ITCH	Downregulated in tissues	To inhibit cell proliferation	Sponge miR-7 and miR-214; upregulate ITCH	Unknown	[21]
Hsa_circ_0043256	Upregulated in NSCLC cells in response to CA treatment	To inhibit cell proliferation and induce apoptosis	Sponge miR-1252; upregulate ITCH; inhibits the Wnt/β-catenin pathway	Unknown	[47]
Circ-BANP	Upregulated in tissues	To promote cell proliferation, migration, and invasion	Sponge miR-503; upregulate LARP1 expression	Higher expression level associated with lower overall survival	[36]
Circ_0067934	Upregulated in NSCLC tissues and cell lines	To promote cell proliferation, migration, and invasion	Modulate the expression levels of markers of epithelial-to-mesenchymal transition	Higher expression level associated with lower overall survival	[43]
CircRNF13	Downregulated in tissues	To inhibit cell invasion and metastasis	Sponge miR-93-5p	Unknown	[8]
CircMAN2B2	Upregulated in tissues and cell lines	To promote cell proliferation and invasion	Sponge miR-1275; upregulate FOXK1 expression	Unknown	[34]
F-circEA	Existence in plasma and H2228 cells	To promote cell migration and invasion	Unknown	F-circEA was specifically existed in EML4-ALK-positive NSCLC	[39]
Hsa_circRNA_103809	Upregulated in tissues and cell lines	To promote cell proliferation and invasion	Sponge miR-4302; upregulate the expression of ZNF121; enhance MYC protein level	Higher expression level associated with lower overall survival	[29]
CircUBAP2	Upregulated in tissues	To promote cell proliferation, invasion and prevent apoptosis	Upregulate CDK6,cyclin D1, c-IAP1, Bcl-2, Survivin, FAK, Rac1, MMP2, JNK and ERK1/2; downregulate p27 and Bax; sponge miR-339-5p, miR-96-3p and miR-135b-3p	Unknown	[28]
Hsa_circ_0000064	Upregulated in tissues and cell lines	To promote cell proliferation and cycle progression and prevent apoptosis	Upregulate caspase-3, caspase-9, bax, p21, CDK6, cyclin D1, MMP-2, and MMP-9; downregulate bcl-2	Unknown	[42]
Hsa_circ_0046264	Downregulated in tissues and cell lines	To inhibit proliferation and invasion, to promote cell apoptosis	Sponge miR-1245; upregulate BRCA2	Lower expression level associated with worse prognosis outcome	[37]
Hsa_circ_0079530	Upregulated in tissues and cell lines	To promote cell proliferation, migration, invasion and cycle progression	Unknown	The area under the ROC curve was 0.756	[41]
CircRNA-FOXO3	Downregulated in tissues and cell lines	To inhibit cell proliferation, migration, and invasion	Sponge miR-155; upregulate FOXO3	The area under the ROC curve was 0.782	[33]
Hsa_circ_0012673	Upregulated in tissues	To promote cell proliferation	Sponge miR-22; upregulate ErbB3	Unknown	[32]
Hsa_circRNA_103827	Upregulated in tissues	Unknown	Unknown	Higher expression level associated with shorter overall survival	[45]
Hsa_circRNA_000122	Downregulated in tissues	Unknown	Unknown	Lower expression level associated with shorter overall survival	[45]
Hsa_circ_0014130	Upregulated in tissues	Unknown	Unknown	The area under the ROC curve was 0.878	[22]
Hsa_circ_0007385	Upregulated in tissues and cells	To promote cell proliferation, migration, and invasion	Sponge miR-181	Unknown	[35]
Circ0006916	Downregulated in 16HBE-T,A549 and H460 cell lines	To inhibit cell proliferation and cycle progression	Sponge miR-522-3p; upregulate PHLPP1	Unknown	[30]
CircPRKCI	Upregulated in tissues	To promote cell proliferation and migration	Sponge miR-545 and miR-589; upregulate E2F7 expression	Higher expression level associated with shorter overall survival	[31]
Circ_001569	Upregulated in tissues	Promote cell proliferation	Promote the Wnt/β-catenin pathway	Higher expression level associated with poorer survival outcome	[44]

#### The function and regulation mechanisms of circRNAs in lung cancer

CDR1as is an antisense transcript of CDR1 [7]. CDR1as contains 63 miR-7 binding sites. Once bound to miR-7, CDR1as inhibits miR-7 function and thus exerts a negative regulatory effect. Based on the role of CDR1as as a miR-7 “sponge,” Hansen et al. hypothesized that the CDR1as/miR-7 axis might be potentially involved in tumorigenesis [18]. MiR-7 downregulates the expression of epidermal growth factor receptor (EGFR) mRNA and protein and participates in tumor regulation as a regulator of EGFR [19]. EGFR activates miR-7 through the Ras/extracellular signal-regulated kinase (ERK)/Myc pathway and inhibits ETS2 repressor factor (ERF, a transcriptional repressor of V-ets avian erythroblastosis virus E26 oncogene homolog 2 (Ets2)), thereby promoting cell growth and the occurrence of lung cancers [20]. A regulatory loop may exist between miR-7 and EGFR. The direction of the balance between miR-7 and EGFR determines whether a cell is benign or malignant [20]. In lung adenocarcinoma tissues with an EGFR mutation, the mutation enhances the expression of miR-7 through promoting EGFR phosphorylation. There is a loss of balance between EGFR and miR-7. EGFR expression levels are significantly positively correlated with miR-7 expression levels [20]. By virtue of its sponge-like adsorptive activity, CDR1as may participate in the regulation of miR-7/EGFR and thus be involved in the development and progression of lung cancers.

**Fig. 1:**
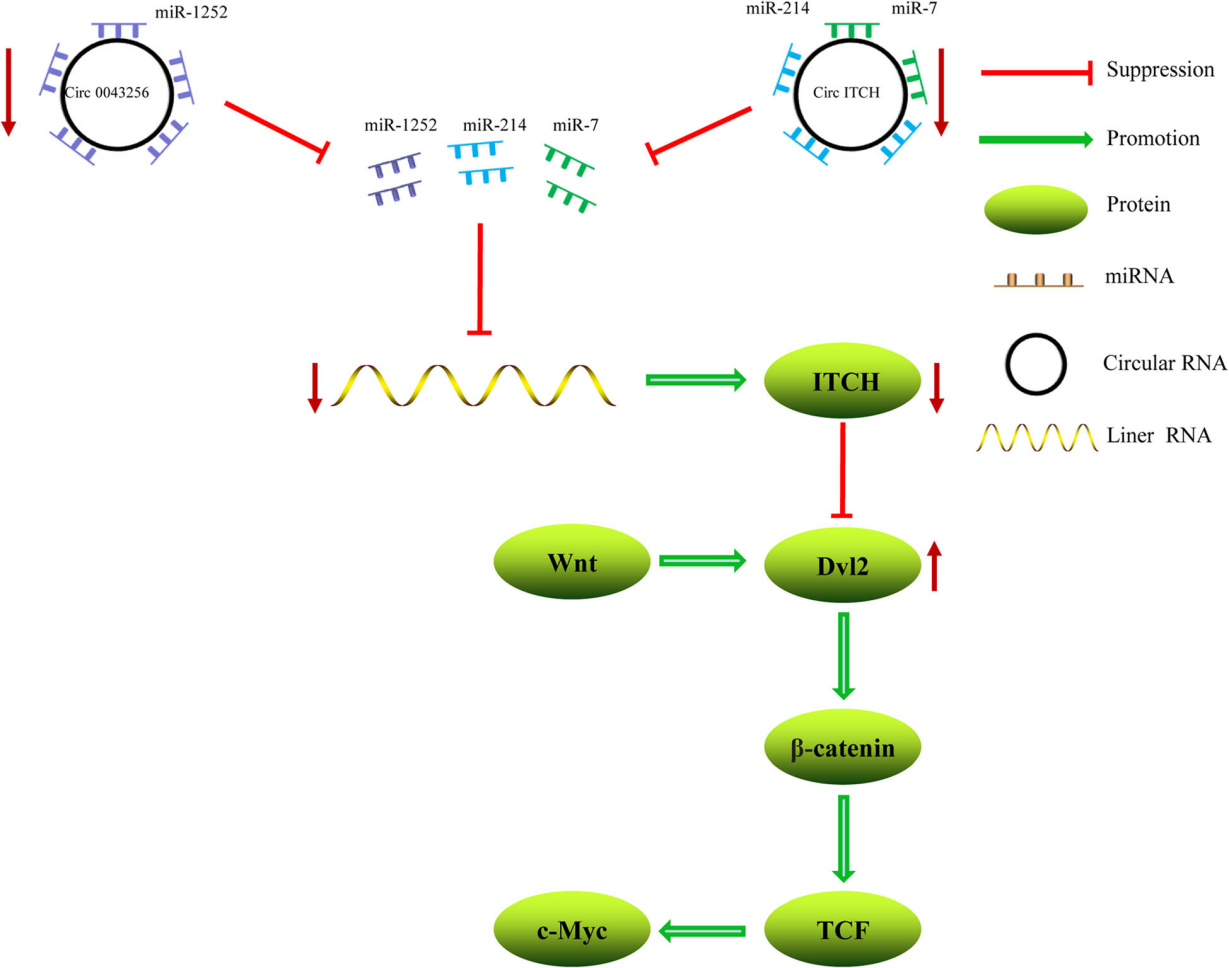
Circular RNA ITCH and 0043256 act as miRNA sponges to regulate the expression of ITCH. Through downregulating the ITCH expression, the Wnt/β-catenin pathway is enhanced

As a circRNA, cir-ITCH and the three prime untranslated regions (3′-UTR) of E3 ubiquitin ligases (ITCH) share some common miRNA binding sites. Cir-ITCH acts as a sponge to absorb miR-7 and miR-214, thereby regulating the expression of ITCH [21]. ITCH participates in the ubiquitin-mediated degradation of a variety of proteins in vivo. ITCH inhibits the Wnt/β-catenin signaling pathway through ubiquitination and degradation of phosphorylated dishevelled-2 (Dvl2). A decrease in cir-ITCH expression allows increased binding of miR-7 and miR-214 to ITCH, which leads to the downregulation of ITCH expression (Fig. 1). The Wnt/β-catenin pathway is thus enhanced, promoting the development, progression, and metastasis of lung cancers. As a miR-1252 sponge, hsa_circ_0043256 binds competitively to miR-1252, thereby affecting the important negative regulator of the Wnt signaling pathway (the E3 ubiquitin ligases ITCH) [22]. Hsa_circ_0043256 upregulates ITCH expression, whereas miR-1252 partially counteracts the upregulatory effect of hsa_circ_0043256. The hsa_circ_0043256/miR-1252/ITCH axis plays an important role in cinnamaldehyde-mediated anti-tumor activity, which provides a new target for the treatment of lung cancer (Fig. 1).

CircHIPK3, a circRNA derived from the homeodomain-interacting protein kinase 3 (HIPK3) gene, and the mRNA of insulin-like growth factor 1 (IGF1) bind jointly to miR-379. CircHIPK3 regulates IGF1 expression through sponging miR-379 [23]. As a highly abundant circRNA, circHIPK3 has 18 potential miRNA binding sites [24]. In HuH-7, HCT-116, and HeLa human tumor cell lines, knockdown of circHIPK3 significantly inhibited tumor cell growth. Among six non-small cell lung cancer (NSCLC) cell lines, H1299 expressed the lowest level of circHIPK3, whereas H2170 expressed the highest level of circHIPK3. Overexpression of circHIPK3 in the NSCLC cell line NCI-H1299 significantly promoted cell proliferation, whereas interference with circHIPK3 expression in NCI-H2170 cells drastically inhibited cell proliferation [23]. circHIPK3 exerts a proliferation-promoting activity in lung cancer cell lines, indicating that it participates in the regulation of lung cancer and may become a new target for the treatment of NSCLC.

*Sry*, a circular transcript of the sex-determining region of Y-chromosome, has 16 conserved binding sites for miR-138. In small cell lung cancer cells, miR-138 targets H2A histone family member X (H2AX) and regulates DNA damage responses. A study found that miR-138 expression was significantly downregulated in small cell lung cancer tissues and cell lines. Overexpression of miR-138 in small cell lung cancer cell lines led to significantly decreased cell proliferation and cell cycle arrest. H2AX knockout induced a cell inhibitory effect similar to miR-138 overexpression. It has been hypothesized that miR-138 targets H2AX and affects the proliferation and cell cycle progression of small cell lung cancer cells through inhibiting H2AX expression [25]. The Sry/miR138/H2AX axis may be involved in the regulation of small cell lung cancer, thereby possessing potential diagnostic and therapeutic intervention values.

CircRNA 100876 and the 3-UTR of matrix metalloproteinase 13 (MMP13) share common miRNA binding sites and bind jointly to miR-136 [26]. Inhibition of circRNA 100876 with small interfering RNA (siRNA) reduces the expression of MMP13, indicating that circRNA 100876 may indirectly regulate MMP13 through sponging miR-136. MMP13 is a member of the matrix metalloproteinase family that promotes lung cancer invasion and metastasis by degrading the extracellular matrix (ECM). Hsa_circ_0013958 acts as a sponge to adsorb miR-134 and upregulates the proto-oncogene cyclin D1, thereby promoting the proliferation and invasion of lung cancer cells [27]. It is likely that circRNA 100876 and circRNA 0013958 exert their carcinogenic effects through affecting the expression of lung cancer-related genes.

CircUBAP2 is highly expressed in lung adenocarcinoma, which is involved in tumor formation, invasion, and metastasis [28]. The results of luciferase reporter assay showed that miR-339-5p, miR-96-3p, and miR-135b-3p could act directly on circUBAP2. By analyzing the expression levels of cell proliferation and apoptosis-associated proteins (CDK6, cyclin D1, p27, c-IAP1, Bcl-2, Survivin, and Bax), it was found that CDK6, cyclin D1, and c-IAP1 expressions were downregulated, while p27 and Bax expressions were increased. CircUBAP2 silencing can inhibit the expression of Rac1 and FAK, further inhibiting the expression of MMP-2. CircUBAP2 silencing can also inhibit JNK and ERK1/2 activity. It is suggested that circUBAP2 may regulate the invasion and metastasis of tumor cells through the Rac-FAK signaling pathway and JNK signaling pathway.

Hsa_circRNA_103809 participates in the regulation of lung cancer through the miR-4302/ZNF121/MYC pathway [29]. Hsa_circRNA_103809 can promote the proliferation and invasion of lung cancer cells. Bioinformatics analysis reveals that hsa_circRNA_103809 can bind to miR-4302, while ZNF121 is the downstream target gene of miR-4302. Luciferase reporter assay showed that miR-4302 could interact with hsa_circRNA_103809 and ZNF-121. Quantitative RT-PCR further confirmed that hsa_circRNA_103809 knockdown significantly promoted miR-4302 expression, while miR-4302 overexpression inhibited ZNF121 expression. It is suggested that hsa_circRNA_103809 regulates ZNF121 expression through acting as a sponge for miR-124. The co-immunoprecipitation assay and western blot experiment further confirmed that ZNF121 can interact with MYC directly. The changes of hsa_circRNA_103809 and ZNF121 have corresponding effects on the level of MYC expression. The research of hsa_circRNA_103809 provides a new way to understand the pathogenesis of lung cancer.

Circ0006916 participates in tumor regulation through the miR-522-3P/PHLPP1 pathway [30]. Circ0006916 is downregulated in 16HBE-T, A549, and H460 cells, which inhibits cell proliferation. Luciferase reporter assay showed that circ0006916 could bind to miR-522-3P. The target gene PHLPP1 of miR-522-3P showed the same changes in the levels of mRNA and protein after the overexpression and downregulation of circ0006916. The results indicate that circ0006916 might affect the expression of PHLPP1 through miR-522-3P. The RNA pull-down experiment confirmed that RNA-binding protein TNRC6A could bind to the flanked intron region of circ0006916, and the absence of TNRC6A decreases the generation of circ0006916, indicating that TNRC6A could regulate the formation of circ0006916. This study reveals the regulatory protein in the upstream of circ0006916, and TNRC6A may participate in the growth of lung cancer cells by regulating circ0006916.

CircPRKCI participates in tumor regulation through the circPRKCI-miR-545/589-E2F7 pathway [31]. CircPRKCI is overexpressed in lung adenocarcinoma (LAC) tissues, which can promote the proliferation and migration of LAC cells. miRNA pull-down assay showed that circPRKCI could be effectively enriched by miR-589 and miR-545. RIP assay also showed that miR-545, circPRKCI, and miR-589 were effectively pulled down by anti-AGO2 antibodies. The silencing of circPRKCI did not affect the expression of miR-545 or miR-589, indicating that circPRKCI serves as a miRNA sponge, and it did not affect the expression of sponged miRNA. The results of qRT-PCR, pull-down assay, and luciferase reporter assay showed that both miR-545 and miR-589 directly bind to the 3′-UTR of E2F7 and directly downregulated the expression of E2F7, while silencing circPRKCI also caused E2F7 downregulation, which further indicated that circPRKC could regulate E2F7 as a miR-545 and miR-589 sponge.

Hsa_circ_0012673 participates in the proliferation of lung adenocarcinoma through the miR-22/ErbB3 pathway [32]. Hsa_circ_0012673 is overexpressed in lung adenocarcinoma and has the effect of promoting cell proliferation. Luciferase reporter assay showed that miR-22 could reduce luciferase reporter activity. Knocking down hsa_circ_0012673 inhibited cell proliferation, but when miR-22 was silenced, the inhibitory effect of hsa_circ_0012673 on cells vanished. These data indicate that hsa_circ_0012673 promotes proliferation of LAC cells through miR-22. The expression of ErbB3 in tumor tissues of LAC patients was upregulated, and the expression level was positively correlated with hsa_circ_0012673, but negatively correlated with miR-22 expression. Western blot results showed that miR-22 could reduce the expression of ErbB3, while hsa_circ_0012673 increased ErbB3 protein level in LAC cells. This article describes the regulatory role of the hsa_circ_0012673/miR-22/ErbB3 pathway in proliferation and provides a new idea for the diagnosis and treatment of lung adenocarcinoma.

CircRNA-FOXO3 acts as a tumor suppressor through the miR-155/FOXO3 pathway [33]. CircRNA-FOXO3 is lowly expressed in NSCLC tissues and cell lines. Overexpression of circRNA-FOXO3 can significantly inhibit cell proliferation, metastasis, and invasion and promote apoptosis. By detecting the expression level of FOXO3 mRNA, it was found that it was downregulated in NSCLC. Spearman correlation analysis showed that the expression levels of circRNA-FOXO3 and FOXO3 mRNA were positively correlated. Bioinformatics analysis predicts that miR-155 can interact with circRNA-FOXO3 and FOXO3 mRNA. RIP assay showed that AGO2 antibody could precipitate circRNA-FOXO3 and FOXO3 mRNA. After circRNA-FOXO3 overexpression, miR-155 decreased significantly in cells. These results indicate that circRNA-FOXO3 can promote FOXO3 expression by adsorbing miR-155 and act as a tumor suppressor.

CircMAN2B2, hsa_circ_0007385, and circ-BANP were upregulated in lung cancer tissues. Hsa_circ_0046264 was downregulated in lung cancer tissues. They played regulatory roles through the adsorption of miRNAs. CircMAN2B2 can promote the expression of FOXK1 through acting as a miR-1275 sponge [34]. Knocking down circMAN2B2 can significantly inhibit the proliferation and invasion of H1299 and A549 cells, and circMAN2B2 may play a carcinogenic role in lung cancer. Hsa_circ_0007385 has the function of promoting cell proliferation, metastasis, and invasion [35]. Bioinformatics analysis and luciferase reporter assay confirmed that miR-181 can bind to hsa_circ_0007385. Circ-BANP [36] can promote cell invasion, proliferation, and metastasis. Mechanism study indicates that circ-BANP can play a regulatory role in the development of lung cancer through the miR-503/LARP1 signaling pathway. Hsa_circ_0046264 was proved to inhibit proliferation and invasion, induce apoptosis of lung cancer cells, and increase BRCA by adsorbing miR-1245 [37].

#### The diagnostic value of circRNAs in lung cancers

Hsa_circ_0013958 exhibits potential diagnostic value in lung cancer. Zhu et al. performed circRNA microarray to investigate the differently expressed circRNAs between lung adenocarcinoma tissues and paired adjacent non-cancerous tissues [27]. A total of 39 circRNAs were highly expressed in lung adenocarcinoma tissues, whereas 20 circRNAs were expressed at low levels in lung adenocarcinoma tissues. Quantitative polymerase chain reaction (qPCR) confirmed that hsa_circ_0013958 was highly expressed in lung adenocarcinoma tissues, plasma, and cell lines. Moreover, the expression level of hsa_circ_0013958 was related to TNM (tumor, node, and metastasis) stage and lymph node metastasis. The diagnostic accuracy of hsa_circ_0013958 was evaluated. In tissue samples, hsa_circ_0013958 yielded an AUC (the area under the receiver operating characteristic (ROC) curve) of 0.815. The sensitivity and specificity of hsa_circ_0013958 for diagnosing lung adenocarcinoma were 0.755 and 0.796, respectively. Plasma hsa_circ_0013958 had an AUC of 0.794. Moreover, hsa_circ_0013958 displayed superior diagnostic accuracy for advanced lung cancer compared with early-stage lung cancer.

Fusion-circRNA (F-circRNA) can be used as a diagnostic marker for the echinoderm microtubule-associated protein-like 4 (EML4)/anaplastic lymphoma kinase (ALK1) gene fusion mutation in lung cancer. Guarneri et al. found that aberrant chromosomal translocations and chromosomal rearrangements are involved in the occurrence of a variety of tumors [38]. Transcription of genes affected by chromosomal translocations may give rise to a new type of aberrant circRNAs, namely F-circRNAs. F-circRNAs are produced via trans-splicing during RNA editing or maturation. F-circRNAs produced via aberrant chromosomal translocation are involved in tumorigenesis and tumor regulation. As proto-oncogene-related RNAs, F-circRNAs promote cell transformation and tumor formation and play a role in treatment resistance. Lung cancer-associated EML4/ALK1 translocation also gives rise to F-circRNA, which may play an important role in the development and progression of lung cancers and serve as a novel entry point for the diagnosis of lung cancer. Tan et al. tested the EML4-ALK-positive cell line H2228 by RT-qPCR, further clarified the existence of F-circEA (fusion-circRNA from EML4-ALK fusion gene), and found that F-circEA has the function of promoting cell proliferation and migration [39]. Further analysis of the expression of F-circEA in the tumor tissues and blood of EML4-ALK-positive patients showed that F-circEA could exist in the plasma and tumor tissues, while the mRNA of the fusion gene EML4-ALK only existed in the tumor tissue and could not be detected in the plasma. The presence of plasma F-circEA suggests that F-circEA can be used as a diagnostic marker for liquid biopsy. F-circEA has the potential value to diagnose the EML4-ALK fusion gene in NSCLC patients and guide the use of ALK inhibitor crizotinib.

CircFARSA in plasma has a diagnostic value for non-small cell lung cancer. Hang et al. have found that circFARSA is overexpressed in NSCLC tissues and plasma, and the functions of circFARSA are to promote cell migration and invasion [40]. Compared with circFARSA extracted from exosomes, the expression of circFARSA extracted from plasma is higher. It is presumed that cells can release circFARSA into the blood through other ways. By detecting plasma circFARSA from 50 patients with NSCLC and 50 healthy controls, it was found that plasma circFARSA had a diagnostic value and the AUC was 0.71. Plasma circFARSA may be used as a molecular marker for non-invasive detection of non-small cell lung cancer.

Hsa_circ_0014130 and hsa_circ_0079530 were highly expressed in NSCLC tissues. The expression of hsa_circ_0014130 was associated with TNM staging and lymph node metastasis [22]. It had a diagnostic value in differentiating lung cancer tissues and adjacent normal tissues. The area under the ROC curve was 0.878, and the sensitivity and specificity were 87% and 84.8% respectively. The expression level of hsa_circ_0075930 was correlated with tumor size and lymph node metastasis. Its AUC was 0.756, the sensitivity was 76.2%, and the specificity was 72.1% [41].

#### The prognostic value of circRNAs in lung cancers

CircRNA_100876 has a prognostic value in patients with lung cancer. Yao et al. examined 101 NSCLC samples, including 51 squamous cell carcinoma (SCC) samples and 50 adenocarcinoma samples [26]. The expression level of circRNA_100876 in tumor tissues was upregulated with 1.23-fold compared with normal tissues. circRNA_100876 expression level was also positively correlated with lymph node metastasis and the TNM stage of lung cancers, indicating that circRNA_100876 might be involved in the growth, proliferation, and metastasis of tumor cells. Patients expressing high levels of circRNA_100876 exhibited reduced overall survival (OS) compared with patients expressing low levels of circRNA_100876, indicating that circRNA_10087 might serve as a risk factor for predicting the prognosis of patients with NSCLC.

Hsa_circRNA_103809 and hsa_circ_0000064 were overexpressed in lung cancer tissues. Kaplan-Meier survival analysis showed that the lung cancer patients with high expression of hsa_circRNA_103809 had lower overall survival (OS), which could be used as a marker for predicting the prognosis of lung cancer [29]. The expression level of hsa_circ_0000064 is significantly correlated with metastasis and malignancy grade of lung cancer [42], and the potential value in predicting prognosis needs to be further verified by clinical research.

Circ_0067934 and circ_001569 were upregulated in NSCLC tissues. The expression of circ_0067934 is associated with TNM staging, lymph node metastasis, and distant metastasis [43]. Multivariate Cox proportional hazards analysis shows that circ_0067934 is an independent factor for poor prognosis in NSCLC patients. Circ_001569 has the function of promoting cell proliferation [44]. The expression level is related to the degree of tumor differentiation, lymph node metastasis, and TNM staging. The high expression of circ_001569 suggests that the prognosis of the patients is poor, and it may play a regulatory role through the Wnt/β-catenin pathway.

Hsa_circRNA_103827 was highly expressed in lung squamous cell carcinoma, while hsa_circRNA_000122 was lowly expressed [45]. Their expression level was correlated with the overall survival of patients. The PCR method further confirmed that Has_circRNA_404833, has_circRNA_406483, has_circRNA_006411, has_circRNA_401977, and has_circRNA_001640 have differential expression in lung cancer tissues [6], but the relationship between their expression levels and the clinicopathological features of the patients should be further studied.

#### The potential value of circRNAs in the treatment of lung cancers

Silencing circPRKCI can inhibit the growth of xenograft tumor in vitro and exhibit a potential therapeutic value [31]. Lin et al. injected SPC-A1 cells transfected with si-circPRKCI into subcutaneous tissue of nude mice. The tumors derived from cells transfected with si-circPRKCI had a smaller size and lower weight than the control group. Compared with xenografts derived from cell lines, patient-derived tumor xenografts (PDTX) maintain better cell differentiation ability, morphology, and architecture of the original patient tumors. The researchers injected cholesterol-conjugated si-circPRKCI and control siRNA into PDTX. Si-circPRKCI can significantly inhibit the growth of PDTX, indicating that circPRKCI is a promising therapeutic target for LAC. EGFR tyrosine kinase inhibitors (EGFR-TKIs) have been widely used in LAC patients with EGFR mutations. In order to find whether circPRKCI affects the therapeutic effects of EGFR-TKI, proliferation assays were performed. Gefitinib combined with si-circPRKCI was more effective than gefitinib or si-circPRKCI alone, which suggested they had potential synergistic therapeutic effect, showing that circPRKCI is a potential therapeutic target.

## Conclusions

Early diagnosis is an important prerequisite for effective treatment of lung cancer. Regional or distant metastasis of tumor cells is the major reason behind the poor efficacy in treatment of lung cancer patients. Computed tomography (CT) of the chest allows early detection of lung lesions. However, it is difficult to differentiate malignant from benign lesions by CT. Bronchoscopy and percutaneous pulmonary puncture allow the acquisition of pathological tissues. However, invasive procedures are often involved and are associated with certain risks. Examination of lung cancer-related protein markers in exfoliated cells and serum is a non-invasive medical procedure. Given that this procedure has limited sensitivity and specificity, new biomarkers are needed to assist in the clinical diagnosis of lung cancer. Due to their endogenous regulatory functions, closed circular structure, high stability, involvement in lung cancer development and progression, and differential expression in lung cancer tissues, circRNAs have the potential to serve as diagnostic and prognostic biomarkers for lung cancer. Given that circRNAs can be secreted to the outside of cells through exosomes and extracellular vesicles (EVs) [46] and EVs are present in a variety of body fluids (such as blood, urine, or saliva), analysis of circRNAs in body fluids is conducive to achieving non-invasive detection of tumors. Optimization of circRNA analysis methods will further improve the detection efficiency of lung cancers. With the gradual maturation of artificial circRNA construction and circRNA interference technology, regulation of circRNAs may become possible, which will provide a new approach for the treatment of lung cancers.

## References

[1] Sanger HL, Klotz G, Riesner D, Gross HJ, Kleinschmidt AK (1976). Viroids are single-stranded covalently closed circular RNA molecules existing as highly base-paired rod-like structures. Proc Natl Acad Sci U S A.

[2] Xu T, Wu J, Han P, Zhao Z, Song X (2017). Circular RNA expression profiles and features in human tissues: a study using RNA-seq data. BMC Genomics.

[3] Torre LA, Bray F, Siegel RL, Ferlay J, Lortet-Tieulent J, Jemal A (2015). Global cancer statistics, 2012. CA Cancer J Clin.

[4] Ettinger DS, Wood DE, Aisner DL (2017). Non-small cell lung cancer, version 5.2017, NCCN clinical practice guidelines in oncology. J Natl Compr Cancer Netw.

[5] Ettinger DS, Wood DE, Akerley W (2016). NCCN guidelines insights: non-small cell lung cancer, version 4.2016. J Natl Compr Cancer Netw.

[6] Zhao J, Li L, Wang Q, Han H, Zhan Q, Xu M (2017). CircRNA expression profile in early-stage lung adenocarcinoma patients. Cell Physiol Biochem.

[7] Memczak S, Jens M, Elefsinioti A (2013). Circular RNAs are a large class of animal RNAs with regulatory potency. Nature.

[8] Wang Lin, Liu Suyao, Mao Yuan, Xu Juqing, Yang Shu, Shen Hongyu, Xu Wei, Fan Weifei, Wang Jun (2018). CircRNF13 regulates the invasion and metastasis in lung adenocarcinoma by targeting miR-93-5p. Gene.

[9] Guo JU, Agarwal V, Guo H, Bartel DP (2014). Expanded identification and characterization of mammalian circular RNAs. Genome Biol.

[10] Zhang Y, Zhang XO, Chen T (2013). Circular intronic long noncoding RNAs. Mol Cell.

[11] Li Zhaoyong, Huang Chuan, Bao Chun, Chen Liang, Lin Mei, Wang Xiaolin, Zhong Guolin, Yu Bin, Hu Wanchen, Dai Limin, Zhu Pengfei, Chang Zhaoxia, Wu Qingfa, Zhao Yi, Jia Ya, Xu Ping, Liu Huijie, Shan Ge (2015). Exon-intron circular RNAs regulate transcription in the nucleus. Nature Structural & Molecular Biology.

[12] Du WW, Yang W, Liu E, Yang Z, Dhaliwal P, Yang BB (2016). Foxo3 circular RNA retards cell cycle progression via forming ternary complexes with p21 and CDK2. Nucleic Acids Res.

[13] Du WW, Yang W, Chen Y (2017). Foxo3 circular RNA promotes cardiac senescence by modulating multiple factors associated with stress and senescence responses. Eur Heart J.

[14] Abe N, Hiroshima M, Maruyama H (2013). Rolling circle amplification in a prokaryotic translation system using small circular RNA. Angew Chem (Int Ed Engl).

[15] AbouHaidar MG, Venkataraman S, Golshani A, Liu B, Ahmad T (2014). Novel coding, translation, and gene expression of a replicating covalently closed circular RNA of 220 nt. Proc Natl Acad Sci U S A.

[16] Chen X, Han P, Zhou T, Guo X, Song X, Li Y (2016). circRNADb: a comprehensive database for human circular RNAs with protein-coding annotations. Sci Rep.

[17] Yang Y, Gao X, Zhang M, et al. Novel role of FBXW7 circular RNA in repressing glioma tumorigenesis. J Natl Cancer Inst. 2018;110(3):304–15. 10.1093/jnci/djx166 PMC6019044 28903484

[18] Hansen TB, Kjems J, Damgaard CK (2013). Circular RNA and miR-7 in cancer. Cancer Res.

[19] Webster RJ, Giles KM, Price KJ, Zhang PM, Mattick JS, Leedman PJ (2009). Regulation of epidermal growth factor receptor signaling in human cancer cells by microRNA-7. J Biol Chem.

[20] Chou YT, Lin HH, Lien YC (2010). EGFR promotes lung tumorigenesis by activating miR-7 through a Ras/ERK/Myc pathway that targets the Ets2 transcriptional repressor ERF. Cancer Res.

[21] Wan L, Zhang L, Fan K, Cheng ZX, Sun QC, Wang JJ (2016). Circular RNA-ITCH suppresses lung cancer proliferation via inhibiting the Wnt/beta-catenin pathway. Biomed Res Int.

[22] Zhang S, Zeng X, Ding T (2018). Microarray profile of circular RNAs identifies hsa_circ_0014130 as a new circular RNA biomarker in non-small cell lung cancer. Sci Rep.

[23] Tian F, Wang Y, Xiao Z, Zhu X (2017). Circular RNA CircHIPK3 promotes NCI-H1299 and NCI-H2170 cell proliferation through miR-379 and its target IGF1. Zhongguo Fei Ai Za Zhi.

[24] Zheng Q, Bao C, Guo W (2016). Circular RNA profiling reveals an abundant circHIPK3 that regulates cell growth by sponging multiple miRNAs. Nat Commun.

[25] Yang H, Luo J, Liu Z, Zhou R, Luo H (2015). MicroRNA-138 regulates DNA damage response in small cell lung cancer cells by directly targeting H2AX. Cancer Investig.

[26] Yao JT, Zhao SH, Liu QP (2017). Over-expression of CircRNA_100876 in non-small cell lung cancer and its prognostic value. Pathol Res Pract.

[27] Zhu X, Wang X, Wei S (2017). hsa_circ_0013958: a circular RNA and potential novel biomarker for lung adenocarcinoma. FEBS J.

[28] Yin Y, Gao H, Guo J, Gao Y (2017). Effect of circular RNA UBAP2 silencing on proliferation and invasion of human lung cancer A549 cells and its mechanism. Zhongguo Fei Ai Za Zhi.

[29] Liu W, Ma W, Yuan Y, Zhang Y, Sun S (2018). Circular RNA hsa_circRNA_103809 promotes lung cancer progression via facilitating ZNF121-dependent MYC expression by sequestering miR-4302. Biochem Biophys Res Commun.

[30] Dai Xin, Zhang Nan, Cheng Ying, Yang Ti, Chen Yingnan, Liu Zhenzhong, Wang Zhishan, Yang Chengfeng, Jiang Yiguo (2018). RNA-binding protein trinucleotide repeat-containing 6A regulates the formation of circular RNA circ0006916, with important functions in lung cancer cells. Carcinogenesis.

[31] Qiu M, Xia W, Chen R (2018). The circular RNA circPRKCI promotes tumor growth in lung adenocarcinoma. Cancer Res.

[32] Wang X, Zhu X, Zhang H (2018). Increased circular RNA hsa_circ_0012673 acts as a sponge of miR-22 to promote lung adenocarcinoma proliferation. Biochem Biophys Res Commun.

[33] Zhang Y, Zhao H, Zhang L (2018). Identification of the tumorsuppressive function of circular RNA FOXO3 in nonsmall cell lung cancer through sponging miR155. Mol Med Rep.

[34] Ma X, Yang X, Bao W (2018). Circular RNA circMAN2B2 facilitates lung cancer cell proliferation and invasion via miR-1275/FOXK1 axis. Biochem Biophys Res Commun.

[35] Jiang MM, Mai ZT, Wan SZ (2018). Microarray profiles reveal that circular RNA hsa_circ_0007385 functions as an oncogene in non-small cell lung cancer tumorigenesis. J Cancer Res Clin Oncol.

[36] Han Jingquan, Zhao Guibin, Ma Xiao, Dong Qing, Zhang Hang, Wang Yue, Cui Jian (2018). CircRNA circ-BANP-mediated miR-503/LARP1 signaling contributes to lung cancer progression. Biochemical and Biophysical Research Communications.

[37] Yang L, Wang J, Fan Y, Yu K, Jiao B, Su X (2018). Hsa_circ_0046264 up-regulated BRCA2 to suppress lung cancer through targeting hsa-miR-1245. Respir Res.

[38] Guarnerio J, Bezzi M, Jeong JC (2016). Oncogenic role of fusion-circRNAs derived from cancer-associated chromosomal translocations. Cell.

[39] Tan S, Gou Q, Pu W (2018). Circular RNA F-circEA produced from EML4-ALK fusion gene as a novel liquid biopsy biomarker for non-small cell lung cancer. Cell Res.

[40] Hang D, Zhou J, Qin N (2018). A novel plasma circular RNA circFARSA is a potential biomarker for non-small cell lung cancer. Cancer Med.

[41] Li J, Wang J, Chen Z, Chen Y, Jin M (2018). Hsa_circ_0079530 promotes cell proliferation and invasion in non-small cell lung cancer. Gene.

[42] Luo YH, Zhu XZ, Huang KW (2017). Emerging roles of circular RNA hsa_circ_0000064 in the proliferation and metastasis of lung cancer. Biomed Pharmacother.

[43] Wang J, Li H (2018). CircRNA circ_0067934 silencing inhibits the proliferation, migration and invasion of NSCLC cells and correlates with unfavorable prognosis in NSCLC. Eur Rev Med Pharmacol Sci.

[44] Ding L, Yao W, Lu J, Gong J, Zhang X (2018). Upregulation of circ_001569 predicts poor prognosis and promotes cell proliferation in non-small cell lung cancer by regulating the Wnt/beta-catenin pathway. Oncol Lett.

[45] Xu J, Shu Y, Xu T (2018). Microarray expression profiling and bioinformatics analysis of circular RNA expression in lung squamous cell carcinoma. Am J Transl Res.

[46] Lasda E, Parker R (2016). Circular RNAs co-precipitate with extracellular vesicles: a possible mechanism for circRNA clearance. PLoS One.

[47] Tian Fang, Yu C.T., Ye W.D., Wang Qian (2017). Cinnamaldehyde induces cell apoptosis mediated by a novel circular RNA hsa_circ_0043256 in non-small cell lung cancer. Biochemical and Biophysical Research Communications.

